# Supplementary material for: Strategy for Use of Genome-Wide Non-Invasive Prenatal Testing for Rare Autosomal Aneuploidies and Unbalanced Structural Chromosomal Anomalies
Source: J Clin Med. 2020 Aug 1;9(8):2466. doi: 10.3390/jcm9082466 (PMC7464024; doi:10.3390/jcm9082466)
Supplement: Supplementary file 1 [file jcm-09-02466-s001.pdf]

Supplemental Materials

Title: Strategy for use of genome-wide noninvasive prenatal testing for rare autosomal aneuploidies and unbalanced structural chromosomal anomalies

Authors: Dr. Pascale Kleinfinger MD; Dr Laurence Lohmann MD; Dr. Armelle Luscan MD ; Dr. Detlef Trost MD; Dr Laurent Bidat MD; Dr. Véronique Debarge MD,PhD; Dr. Vanina Castaigne MD; Dr. Marie-Victoire Senat MD,PhD; Dr. Marie-Pierre Brechard MD; Ms. Lucie Guilbauld; Dr. Gwenaël Le Guyader MD; Dr. Véronique Satre MD,PhD ; Dr. Hélène Laurichesse Delmas MD, PhD; Dr. Hakima Lallaoui MD; Dr. Marie-Christine Manca-Pellissier MD, Dr. Aicha Boughalem MD, Dr Mylene Valduga, Ms. Farah Hodeib, Dr. Alexandra Benachi MD, PhD; Dr. Jean Marc Costa MD

Table S1. Overview of the 42 abnormal samples in Cohort A.

Sample	Karyotype/array result	Indication for invasive procedure	Tissue type	Array region size (Mb)	GA (weeks)	FF (%)	NIPT call	NIPT result
1	CVS: 47,XX,+16<br>AF : 46,XX	MSS 1/24	CVS/AF	n/a	21.6	19	Trisomy 16	TP
2	CVS-D: 47,XX,+16<br>CVS-C: 47,XX,+16[10]/46,XX[18]<br>AF : 46,XX	MSS 1/58	CVS/AF	n/a	16.4	13	Trisomy 16	TP
3	CVS-D and CVS-C: 47,XY,+16<br>AF : 46,XY	MSS 1/10	CVS/AF	n/a	17.4	18	Trisomy 16	TP
4	47,XX,+22	NC	AF	n/a	23	14	Trisomy 22	TP
5	46,XY,dup(2)(p11p16.1)dn.arr[GRCh37]<br>2p16.1p11.2(56883079_86784499)x3	NC	CVS/FB	29.9	20	15	dup(2)(p16.1p11.2)	TP

6	46,XX,del(4)(p16.3).ish del(4)(WHS-,D4S3359-)	MSS 1/227	CVS	n/a	19.2	10	No anomaly	FN
7	46,XY,del(4)(q13q22).arr[GRCh37] 4q13.3q22.3(70954244_97557598)x1	UA	AF	26.6	31.2	12	del(4)(q13.3q22.3)	TP
8	46,XX,del(4)(p15.1)dn	UA	AF	n/a	35.5	17	del(4)(p16.3p15.2)	TP
9	46,XX,add(4)(qter).ish add(4)(wcp4-).arr[GRCh37] 5q31.2q35.3(138522878_180715096)x3	MSS 1/29	CVS	41.2	19	3	No anomaly	FN
10	46,XX,del(10)(p13p14)dn	NC	AF	n/a	21	10	del(10)(p14p12.31)	TP
11	arr[GRCh37] 10q21.2q22.1(63245223_72075649)x1	UA	CVS	8.8	17.5	8	del(10)(q21.2q22.1)	TP
12	46,XY,del(21)(q22).arr[GRCh37] 21q22.13q22.3(38238871_44968648)x1	UA	AF	6.7	22.6	19	del(21)(q22.2q22.3)	TP
13	46,XY,der(4)t(3;4)(q27;p16.3).arr[GRCh37] 3q27q29(186358366_197838262)x3, 4p16.3p15.32(71,566_17472734)x1	UA	CVS	11.5/17.4	12.3	10	dup(3)(q27.3q29); del(4)(p16.3p15.31)	TP
14	46,XX,der(4)t(4;8)(q35;q22)pat	PT	AF	n/a	21.6	9	dup(8)(q21.3q24.3)	TP

15	46,XY,der(4)t(4;12)(p16.1;q24.21)mat	PT	AF	n/a	23.1	6	dup(12)(q24.21q24.33)	TP
16	46,XY,der(4)t(4;14)(q32.3;q31.3)mat	PT	CVS	n/a	13.5	7	del(4)(q32.3q35.2)	TP
17	46,XX,der(5)t(3;5)(q26,3;p15,2)mat	MSS 1/120	AF	12.8	20.3	21	dup(3)(q26.33q29);<br>del(5)(p15.33p15.2)	TP
18	46,XY,der(5)t(5;13)(p13.3;q12.2)mat	UA	AF	n/a	21	10	del(5)(p15.33p13.3);<br>dup(13)(q21.33q34)	TP
19	46,XY,der(6)t(6;7)(p25;q21)	MSS 1/20	AF	n/a	23.2	6	dup(7)(q22.1q36.2)	TP
20	46,XY,der(6)t(6;11)(q27;q23)mat	PT	CVS	n/a	18.2	8	dup(11)(q23.3q25)	TP
21	46,XY,t(1;3)(p32;q21),der(9)t(4;9)(p14;q34.3)pat	PT	CVS	n/a	13.5	8	dup(4)(p16.3p15.2)	TP
22	46,XX,der(9)t(5;9)(q34;p24.1)pat	UA	CVS	7.9	13.2	6	dup(5)(q34q35.3);<br>del(9)(p24.3p24.1)	TP
23	46,XY,der(11)t(11;12)(p25;q24.31)dn.arr[GRCh37]<br>11q25(132187876-134938470)x1,<br>12q24.31q24.33(122122099- 133777902)x3,	NC	AF	2.7/11.7	21.1	12	Trisomy 11	TP

	Xp22.33p22.32(3480348-5896155)x2							
24	46,XY,der(12)t(10;12)(p11.23;p13.32)mat	NC	CVS	n/a	12.3	13	dup(10)(p15.3p11.23)	TP
25	46,XX,der(13)t(3;13)(q21;q33).arr[GRCh37]<br>3q22.3q29(137909993_197851986)x3,<br>13q34(112331920_115107733)x1	PT	CVS	59.9/2.8	11.3	7	dup(3)(q22.3q29)	TP
26	46,XX,der(13)t(9;13)(p24.3;q34)pat.arr[GRCh37]<br>9p24.3p23(208454_12699776)x3,<br>13q34(110950631_115107733)x1	UA	AF	12.5/4.1	25.3	24	dup(9)(p24.3p23)	TP
27	46,XX,der(13)t(13;18)(q34;q21.1)pat	PT	CVS	n/a	12.6	13	dup(18)(q21.1q23)	TP
28	46,XX,der(15)t(10;15)(p10;p10)pat	MSS 1/60	AF	n/a	19.4	8	dup(10)(p15.3p11.1)	TP
29	46,XX,der(18)t(4;18)(q35;p11.2)pat.arr[GRCh37]<br>4q35.1q35.2(182728284_190916678),<br>18p11.3p11.22(118760_9886069)	PT	CVS	8.2/9.8	12	12	del(18)(p11.32p11.22)	TP
30	46,XY,der(18)t(10;18)(p12;p11.2).arr[GRCh37]<br>10p15.3p12.33(100047_17467979)x3,	NC	CVS	17.3/11.8	17.5	9	del(18)(p11.32p11.21)	TP

	18p11.32p11.21(136227_11938685)x1							
31	46,XX,add(21)(q22?). arr[GRCh37]<br>21q22.11q22.2(35526716_40301704)x3,<br>21q22.2(40608415_42421024)x3,<br>21q22.3(42766413_46101163)<br>x3,21q22.3(46303942_47476920)x3	UA	AF	11.1	22.6	18	dup(21)(q22.12q22.3)	TP
32	46,XX,der(21)t(11;21)(q13;q22)pat	PT	CVS	n/a	14.1	7	dup(11)(q13.5q25)	TP
33	47,XX,+der(22)t(4;22)(p16;q11.2)pat	UA	AF	n/a	24.5	21	dup(4)(p16.3p15.33)	TP
34	47,XY,+der(22)t(11;22)(q23;q11.1)	NC	CVS	n/a	16.2	8	dup(11)(q23.3q25)	TP
35	47,XY,+der(22)t(11;22)(q23;q11.1)	NC	CVS	n/a	13.5	11	dup(11)(q23.3q25)	TP
36	47,XX,+der(22)t(11;22)(q23q11.2).arr[GRCh37]<br>11q23.3q25(116683754_134937416)x3,<br>22q11.1q11.21(16888899_20312661)x3	UA	AF	18.3/3.4	25.1	23	dup(11)(q23.3q25)	TP
37	47,XX,+i(12)(p10)[12]/46,XX[6]	NC	CVS	n/a	14.6	8	dup(12)(p13.33q12)	TP

38	47,XY,+i(12)(p10)	UA	AF	n/a	18.2	11	dup(12)(p13.33p11.21)	TP
39	arr[GRCh37] 12p13.33q11(173786_37876500)x3	UA	AF	37.7	16	15	No anomaly	FN
40	46,XY,i(18)(q10)	NC	CVS	n/a	14.5	4	No anomaly	FN
41	mos 47,XX,+mar[5]/46,XX[24].<br>nuc ish 15q11q13(SNRPNx3)[30/100]	PT*	CVS/AF	n/a	19.1	12	dup(15)(q11.1q14)	TP
42	46,XX,der(8)?add(8)(p?)?dup(8)(q22q23)dn.ish<br>der(8)(qter->?::?->qter)(D8S504-<br>,VIJyRM2053+,wcp8+,VIJyRM2053+).arr[GRCh37]<br>8p23.3p23.1(158048_6935930)x1,<br>8p23.1p11.23(12585435_38267493)x3,<br>8p11.22(38314367_39246760)x3,<br>8p11.22(39247087_39386852)x1,<br>8p11.22(39389765_40264413)x3,<br>8q22.3q23.2(104688373_111952230)x3,<br>8q24.3(144972747_146295771)x3	UA	AF	n/a	27.5	9	No anomaly	FN

AF, amniotic fluid; CVS, chorionic villus sampling (direct and culture); CVS-C, chorionic villus sampling culture; CVS-D, chorionic villus sampling direct; FB, fetal biopsy (post abortion); FF, fetal fraction; FP, false positive; GA, gestational age; MSS, maternal serum screening at first trimester; NC, nuchal translucency ≥3.5mm; PT, parental translocation; PT*, father with a Robertsonian translocation(13:14), TP, true positive; UA, ultrasound anomaly
